# Serum CNPY2 isoform 2 represents a novel biomarker for early detection of colorectal cancer

**DOI:** 10.18632/aging.101512

**Published:** 2018-08-02

**Authors:** Jianhong Peng, Qingjian Ou, Zhizhong Pan, Rongxin Zhang, Yujie Zhao, Yuxiang Deng, Zhenhai Lu, Lin Zhang, Caixia Li, Yaxian Zhou, Jian Guo, Desen Wan, Yujing Fang

**Affiliations:** 1 Department of Colorectal Surgery, Sun Yat-sen University Cancer Center, State Key Laboratory of Oncology in South China, Collaborative Innovation Center for Cancer Medicine, Guangzhou, Guangdong 510060, P. R. China; 2 Department of Experimental Research, Sun Yat-sen University Cancer Center, State Key Laboratory of Oncology in South China; Collaborative Innovation Center for Cancer Medicine, Guangzhou, Guangdong 510060, P. R. China; 3 Department of Clinical Laboratory Medicine, Sun Yat-sen University Cancer Center, State Key Laboratory of Oncology in South China; Collaborative Innovation Center for Cancer Medicine, Guangzhou, Guangdong 510060, P. R. China; 4 School of Mathematics and Computational Science, Sun Yat-sen University, Guangzhou, Guangdong P. R. China; 5 Senboll Biotechnology Co., Ltd., Pingshan Bio-Pharmacy Business Accelerator, Pingshan District, Shenzhen, Guangdong 518000, P. R. China; * Equal contribution

**Keywords:** CNPY2 isoform 2, colorectal cancer, diagnosis, combination

## Abstract

Since early diagnosis is very important for treating CRC, we decided to detect peripheral serum canopy fibroblast growth factor signaling regulator 2 (CNPY2) isoform 2 to verify its diagnostic value for CRC patients. Serum samples were collected from 430 CRC patients and 201 healthy controls. Enzyme-linked immunosorbent assay (ELISA) detection kits for CNPY2 isoform 2 were generated and then applied to measure serum CNPY2 isoform 2 concentrations. Serum carcinoembryonic antigen (CEA) and carbohydrate antigen 19-9 (CA19-9) were also measured. The median serum CNPY2 isoform 2 concentrations in all CRC patients were significantly higher than those in the healthy control group (all P<0.001). Those with stage I CRC presented the highest area under the receiver operating characteristic curve (AUC) for CNPY2 isoform 2 [0.707, 95% confidence interval (CI): 0.649–0.765, P<0.001]. The diagnostic efficiency of the combination of CNPY2 isoform 2, CEA and CA19-9 was significantly higher than that of each biomarker detected separately (all P<0.0167). Serum CNPY2 isoform 2 may be a valuable biomarker for the early detection of CRC and presents an improvement in the diagnostic efficiency by combination of CEA and CA19-9.

## Introduction

Colorectal cancer (CRC), one of the most common newly diagnosed cancers, has been ranked as the third leading cause of cancer-related deaths in China and developed countries [1,2]. Curative treatment for early-stage CRC is usually effective, as surgical resection can achieve a favorable 5-year survival rate that reaches 70-90% [3,4]. However, metastatic CRC presents a poor 5-year survival rate of less than 20% even when surgery and comprehensive treatment have been performed [3,5]. Therefore, early detection of CRC is of great importance so that timely treatment can be implemented to avoid the occurrence of metastatic disease.

Colonoscopy examination and biopsy are the two most effective measures to definitively diagnose CRC. However, colonoscopy is an interventional examination, leading to poor compliance by patients, which makes it difficult to widely implement [6,7]. Since peripheral blood can be non-invasively obtained and easily stored, detection of multiple serum biomarkers has become an alternative method for helping to make early diagnoses of CRC. Currently, carcinoembryonic antigen (CEA) and carbohydrate antigen 19-9 (CA19-9) have been widely used for auxiliary CRC diagnosis and prognostic prediction [8,9]. Previous studies have reported that CEA and CA19-9 presented a diagnostic sensitivity of less than 50% [10,11]. This means that more than half of CRC cases will be misdiagnosed on the basis of CEA or CA19-9 alone. Therefore, the need to find more effective serum biomarkers for the early detection of CRC is urgent, as this would help optimize early CRC treatment.

Canopy fibroblast growth factor signaling regulator 2 (CNPY2) belongs to the canopy family of proteins and has two encoded isoforms [12]. Previous study demonstrated that CNPY2 was up-regulated by HIF-1a protein in hypoxia condition and secreted as an angio-genic growth factor, participating in tissue revascularization [13]. Furthermore, CNPY2 promoted CRC and renal cell carcinoma progression by enhancing cell proliferation, migration, and angiogenesis and inhibiting apoptosis through upregulation of the p53 pathway [14,15]. CNPY2 isoform 1 is a long-chain protein with 182 amino acids, is widely expressed in multiple organs and tissues and detectable in the serum of cancer patients [15,16]. CNPY2 isoform 2 was identified as another CNPY2 encoded protein that consisted of only 84 amino acids. Compared to CNPY2 isoform 1, CNPY2 isoform 2 has a homogeneous region in the first 69 amino acids but possesses a different C-terminal. Our previous study first found that CNPY2 isoform 2 was a novel biomarker that is highly expressed in the cytoplasm of CRC cells and associated with CRC prognosis [17]. Based on an amino acid sequence analysis, CNPY2 isoform 2 has a signal peptide in the first 20 amino acids and does not contain any transmembrane domains, suggesting that it functions as a secreted protein. To date, data on human serum levels of CNPY2 isoform 2 are still lacking. Whether it is detectable in peripheral blood and the value of measuring this protein for the early diagnosis of CRC in patients have not yet been confirmed. Hence, CNPY2 isoform 2 requires further investigation to determine its potential diagnostic value.

In this study, we measured CNPY2 isoform 2 concentrations in 631 serum samples by enzyme-linked immunosorbent assay (ELISA). We aimed to confirm (1) if CNPY2 isoform 2 is detectable in peripheral blood, both in CRC patients and healthy control subjects; (2) if there are differences in the serum levels of CNPY2 isoform 2 between CRC patients and healthy controls; (3) if it is feasible to apply CNPY2 isoform 2 measurements for the early detection of CRC; and (4) if the combination of CNPY2 isoform 2, CEA and CA19-9 results in an improved efficacy for CRC diagnosis.

## RESULTS

### Median serum levels of CNPY2 isoform 2 in the healthy controls and patients prior to surgery

The median age of the 201 healthy controls was 35 (range 19-88) years, with 107 males and 94 females. The median absolute serum CNPY2 isoform 2 concentration was 4.32 (0–36.36) pg/ml in healthy controls. The median age of the 430 CRC patients was 59 (range 15-91) years, with 249 males and 181 females. There were 107 patients with stage I, 107 patients with stage II, 108 patients with stage III and 108 patients with stage IV disease. The median absolute serum CNPY2 isoform 2 concentration was 6.97 (range 0–41.91) pg/ml in all patients, 7.13 (range 1.45–26.64) pg/ml in stage I patients, 6.83 (range 0.12–41.91) pg/ml in stage II patients, 7.32 (range 0.16–38.65) pg/ml in stage III patients and 6.23 (range 0–39.89) pg/ml in stage IV patients.

**Figure 1:**
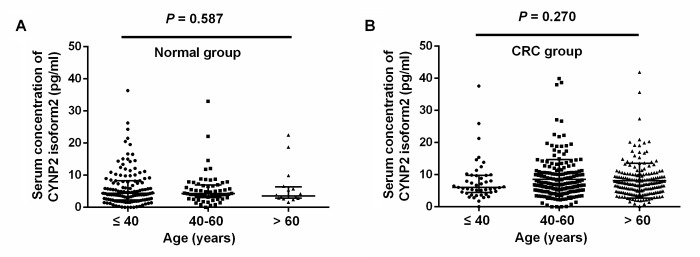
**Scatter plots of serum CNPY2 isoform 2 concentrations among different age groups.** (**A**) Comparison of serum CNPY2 isoform 2 levels of different age groups in healthy controls. (**B**) Comparison of serum CNPY2 isoform 2 levels of different age groups of colorectal cancer (CRC) patients. A Mann–Whiney U test was used to compare the CNPY2 isoform 2 levels among different age groups.

In healthy controls group, the median serum CNPY2 isoform 2 concentration in age≤40 years, age 40 -60 years, and age > 60 years were 4.40 (range 0.00–36.36) pg/ml, 4.29 (range 0.00–33.00) pg/ml, and 3.56 (range 1.60–22.51) pg/ml, which were not significantly different (*P*=0.587, Figure 1A). In CRC group, the median serum CNPY2 isoform 2 concentration in age≤40 years, age 40 -60 years, and age > 60 years were 6.03 (range 0.00–37.60) pg/ml, 6.85 (range 0.00–39.89) pg/ml, and 7.24 (range 0.16–41.91) pg/ml, which were also comparable (*P*=0.270, Figure 1B).

**Figure 2:**
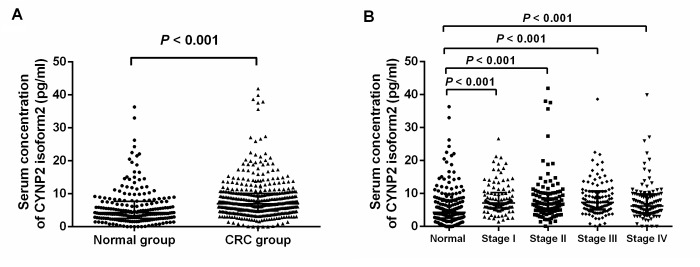
**Scatter plots of serum CNPY2 isoform 2 concentrations in healthy controls and colorectal cancer (CRC) patients.** (**A**) Comparison of serum CNPY2 isoform 2 levels between all patients and healthy controls. (**B**) Comparison of serum CNPY2 isoform 2 levels between patients at different stage and healthy controls. A Mann–Whiney U test was used to compare the CNPY2 isoform 2 levels between the two groups.

The median serum CNPY2 isoform 2 concentration in all CRC patients were significantly higher than that in healthy controls (P<0.001, Figure 2A). Similarly, the median serum CNPY2 isoform 2 concentrations in patients at each individual stage (I-IV) were significantly higher than those in healthy controls (both P<0.001, Figure 2B). However, there was no significant difference in the median serum CNPY2 isoform 2 levels among patients at different stages (P=0.387).

### Associations between serum levels of CNPY2 isoform 2 and clinical pathological characteristics

Comparisons of median serum levels of CNPY2 isoform 2 in different subgroups with respect to sex, age, tumor location, tumor size, histological type, and tumor-node-metastasis (TNM) stage were performed among all 430 CRC patients. The results indicated that CNPY2 isoform 2 concentration was not correlated with any of these clinical pathological characteristics in CRC patients (all *P*>0.05) (Table 1).

**Table 1:** Level of serum CNPY2 isoform 2 in relation to different clinicopathologic characteristics of colorectal cancer patients.

**Variable**	**No.(%)**	**Median(IQR25–75)**	***P* value**
Total	430	6.97(4.76-10.13)	
Sex			0.201
Male	249(57.9)	7.01(4.55-9.86)	
Female	181(42.1)	6.64(5.10-11.03)	
Age (year)			0.952
≤60	242(56.3)	6.73(4.80-10.30)	
>60	188(43.7)	7.24(4.62-9.97)	
Location			0.345
Right side of colon	148(34.4)	6.84(4.05-9.83)	
Left side of colon	203(47.2)	6.87(5.16-10.42)	
Rectum	79(18.4)	7.03(5.15-10.23)	
Tumor size (cm)			0.183
≤4	268(62.3)	7.04(4.82-10.58)	
>4	162(37.7)	6.69(4.62-9.40)	
Histological type			0.572
Well/moderately	315(73.3)	6.98(4.80-10.23)	
Poorly/mucinous	115 (26.7)	6.90(4.76-9.78)	
T stage			0.386
1-2	111(319)	7.01(5.49-10.12)	
3-4	319(74.2)	6.90(4.53-10.14)	
N stage			0.885
0	243(56.5)	6.98(4.91-10.12)	
1-2	187(43.5)	6.96(4.62-10.23)	
TNM stage			0.664
I-III	322(74.9)	7.04(5.16-10.32)	
IV	108(25.1)	6.23(4.01-9.76)	

Abbreviations: IQR, interquartile range; TNM, tumor-node-metastasis classification

### Quantitative detection of CEA and CA19-9 prior to surgery

The quantitative measured CEA were available for a total of 425 CRC patients and 162 healthy controls. The median serum CEA concentrations were 3.96 (0.31–4935.00) ng/ml in CRC patients and 1.74 (0.37–13.07) ng/ml in healthy controls, which were significantly different (*P* <0.001). The quantitative measurements of CA19-9 were available for 425 CRC patients and 94 healthy controls. The median serum CA19-9 concentrations were 12.67 (0.60–20000.00) U/ml in CRC patients and 8.43 (0.60–38.45) pg/ml in healthy controls, which were also significantly different (P<0.001).

### Diagnostic efficiency of serum CNPY2 isoform 2 in colorectal patients

**Figure 3:**
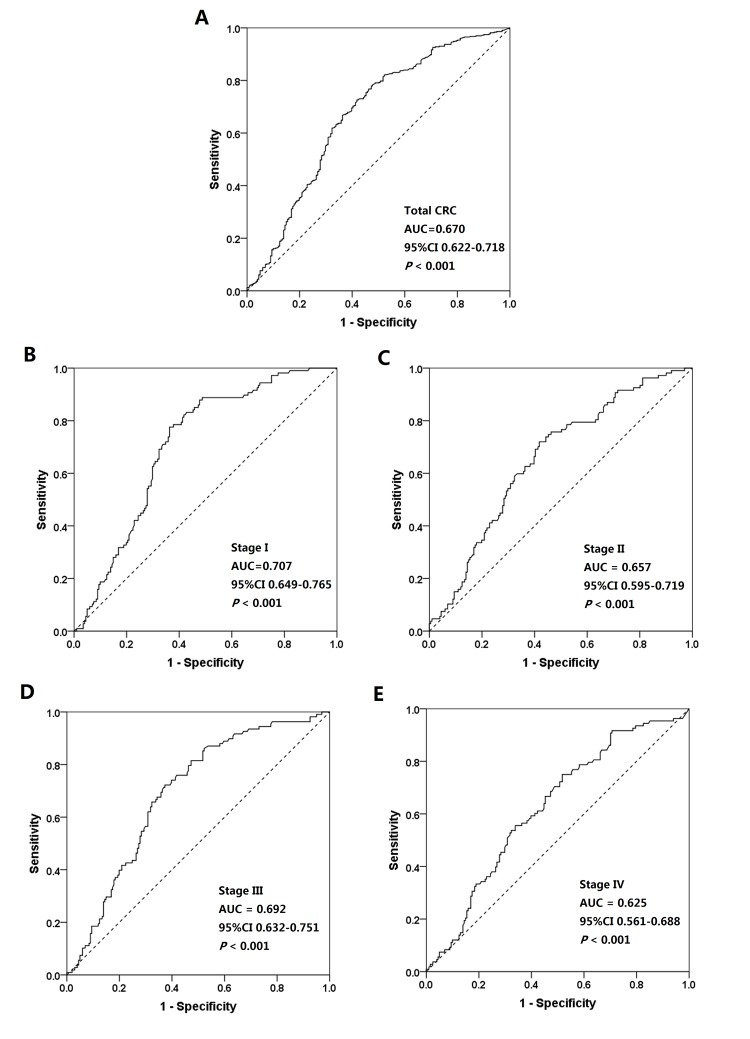
**Receiver operating characteristic (ROC) curves for distinguishing colorectal cancer (CRC) patients from healthy controls.** (**A**) ROC curves for all CRC patients and healthy controls. (**B**) ROC curves for stage I CRC patients and healthy controls. (**C**) ROC curves for stage II CRC patients and healthy controls, (**D**) ROC curves for stage III CRC patients and healthy controls. (**E**) ROC curves for stage IV CRC patients and healthy controls.

Among a total of 430 CRC patients and 201 healthy controls, ROC curve analysis showed that the AUC for distinguishing CRC patients from healthy controls on the basis of serum CNPY2 isoform 2 was 0.670 (95% CI: 0.622–0.718, *P*<0.001) (Figure 3A). The optimal serum CNPY2 isoform 2 cutoff value was 5.00 pg/ml at the highest Youden index of 0.308, with a sensitivity of 72.6% and specificity of 58.2% (Table 2). In stage I-IV patients, the AUCs for distinguishing CRC patients from healthy controls on the basis of serum CNPY2 isoform 2 were 0.707 (95% CI: 0.649–0.765, *P*<0.001), 0.657 (95% CI: 0.595–0.719, P<0.001), 0.692 (95% CI: 0.632–0.751, *P*<0.001) and 0.625 (95% CI: 0.561–0.688, P<0.001), respectively (Figure 3B-D).The sensitivities and specificities of different serum CNPY2 isoform 2 levels for diagnosing stage I-IV patients are shown in Table 2.

**Table 2:** Sensitivities and specificities of serum CNPY2 isoform 2 concentrations for the diagnosis of different stages of colorectal cancer in patients.

Serum CNPY2 isoform 2			Sensitivity (%)				Specificity (%)
concentration (pg/ml)	Total (n=430)	Stage I (n=107)	Stage II (n=107)	Stage III (n=108)	Stage IV (n=108)		
20	4.0	2.8	4.7	2.8	3.7		96.0
10	26.3	28.0	27.1	29.6	20.4		85.1
9	31.9	31.8	32.7	33.3	29.6		82.6
8	39.5	40.2	40.2	42.6	35.2		77.1
7	48.9	54.2	47.7	52.8	44.4		72.1
6	60.0	66.4	57.0	63.9	52.8		67.7
5	72.6	82.2	72.0	75.9	60.2		58.2
4	82.3	88.8	78.5	87.0	75.0		46.2
3	91.2	93.5	87.9	93.5	89.8		29.8

### Diagnostic efficiency of CNPY2 isoform 2, CEA, CA19-9 and their combination

**Figure 4:**
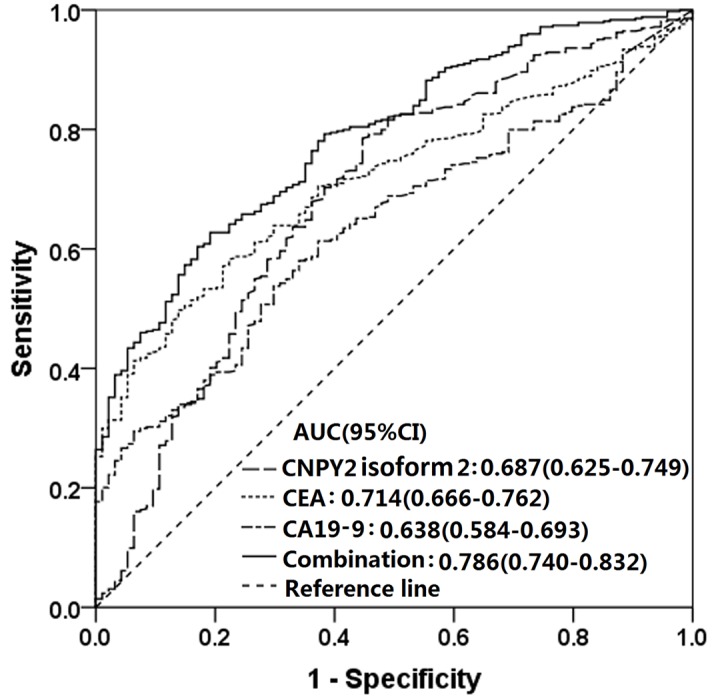
**Receiver operating characteristic (ROC) curves of serum CNPY2 isoform 2, CEA and CA19-9 considered separately and for the combined use of all three in predicting colorectal cancer (CRC).** The area under the ROC curve (AUC) of the combination of all 3 was 0.786 (95% CI :0.740-0.832, P<0.001). The AUC comparison by the DeLongs Algorithm indicated that the AUC of the combined use of all 3 markers was greater than that of each marker detected respectively.

The ROC curve analyses of CNPY2 isoform 2, CEA and CA19-9 individually and of the combination of these 3 markers in 425 CRC patients and 94 healthy controls are shown in Figure 4. The AUCs for CNPY2 isoform 2, CEA and CA19-9 were 0.687(95% CI: 0.625-0.749, *P*<0.001), 0.714(95% CI: 0.666-0.762, *P*<0.001) and 0.638 (95% CI: 0.584-0.693, P<0.001), respectively. The sensitivities and specificities at cutoff values for CNPY2 isoform 2 (5.00 pg/ml), CEA (5.0 ng/ml) and CA19-9 (35.0 U/ml) are shown in Table 3. The AUC for the combination of these 3 markers was 0.786 (95% CI: 0.740-0.832, *P*<0.001), with a sensitivity of 62.7% and a specificity of 81.1% at the highest Youden index of 0.445. The AUC comparison by the DeLongs Algorithm indicated that the AUC for the combination of these markers was greater than that of each marker detected individually (all *P*<0.0167, Table 3).

**Table 3:** Diagnostic value of serum CNPY2 isoform 2, CEA, CA19-9 and the combination of all 3 markers for colorectal cancer patients.

**Markers**	**Sensitivity(%)**	**Specificity(%)**	**AUC**	**95% CI**	***P* value (AUC)**	***P* value** **(comparison with combination of all 3 markers)**
**CNPY2 isoform 2**	72.6	58.5	0.687	0.625-0.749	<0.001	0.001
**CEA**	40.8	93.6	0.714	0.666-0.762	<0.001	<0.001
**CA19-9**	19.8	98.9	0.638	0.584-0.693	<0.001	<0.001
**Combination**	62.7	81.8	0.786	0.740-0.832	<0.001	

Abbreviations: AUC, area under the receiver operating characteristic curve; CI, confidence interval; CEA, carcinoembryonic antigen; CA19-9, carbohydrate antigen 19-9

## DISCUSSION

Recently, CNPY2 isoform 2 was identified as a novel biomarker in oncologic research by examination of its expression in CRC cell lines and tissues [17]. In the current study, we further focused on the detection of serum CNPY2 isoform 2 in CRC patients. Consistent with our previous hypothesis that CNPY2 isoform 2 functions as a secreted protein, CNPY2 isoform 2 were detectable in peripheral blood both in CRC patients and healthy controls. In addition, CNPY2 isoform 2 levels were comparable in different age group both in health control and CRC patients. The serum CNPY2 isoform 2 levels were significantly elevated in CRC patients at various stages compared with those in healthy controls, indicating that serum CNPY2 isoform 2 represents as a potential serum biomarker associated with CRC.

Unexpectedly, high levels of CNPY2 isoform 2 did not indicate a larger tumor size or advanced TNM stage. Similarly, we previously assessed the associations between CNPY2 isoform 2 expression and various clinicopathological parameters, including tumor size and clinical stage. As a result, no significant association was found between CNPY2 isoform 2 expression with the above clinicopathological characteristics [17]. We considered that serum CNPY2 isoform 2 might have been significantly up-regulated in the early stages of tumorigenesis but not in the tumor progression.

It was well known that high sensitivity is an important index in order to avoid false negative diagnosis. Therefore, we determine cutoff value of 5.00 pg/ml at the the highest Youden index of 0.308, with a considerable high sensitivity of 72.6%. Subsequently, we evaluated the diagnostic utility of serum CNPY2 isoform 2 in CRC patients at different stages. ROC curve analysis showed that the AUCs of CNPY2 isoform 2 for distinguishing stage I-IV CRC patients from healthy controls were 0.707, 0.657, 0.692 and 0.625, respectively, indicating that the AUC of stage I CRC patients is greater than those of stage II-IV CRC patients. These results suggested that CNPY2 isoform 2 is more suitable for the early diagnosis of asymptomatic CRC, which has important CRC screening significance. On the other hand, although CNPY2 isoform 2 is elevated in a significant proportion of individuals with advanced CRC, this marker does not appear to be useful alone as a diagnostic tool since the AUCs of CNPY2 isoform 2 for distinguishing stage II-IV CRC were less than 0.700. Previous studies have shown that elevated levels of CEA and CA19-9 were more sensitive for the diagnosis of advanced CRC, because elevated levels of these markers often indicate the occurrence of metastatic disease [18,19]. These results revealed that the diagnostic values of serum CNPY2 isoform 2, CEA and CA19-9 might be complementary in all CRC patients.

Therefore, we combined measurements of serum CNPY2 isoform 2, CEA and CA19-9 in order to improve the diagnostic efficiency for all CRC patients. Ning S et al explored the diagnostic value of the joint detection of thymidine kinase 1 (TK1), CEA, CA199 and carbohydrate antigen 72-4 (CA 72-4) in the diagnosis of gastric cancer (GC) and CRC. They found that the AUC of the combination of these markers was greater than that of each tumor marker detected individually for both GC and CRC, which suggested that the combined detection of these four tumor markers may prove to be useful for the diagnosis of GC and CRC [20]. Wilhelmsen M et al found that a combination of all 8 serological protein biomarkers, including alpha fetoprotein (AFP), CA19-9, CEA, high-sensitivity C-reaction protein (hs-CRP), CyFra21-1, ferritin, galectin-3 and tissue inhibitor of metalloproteinases-1 (TIMP-1), provided a significant improvement for the identification of subjects with a high risk of CRC [21]. Werner S et al tested serum samples from 1660 blood samples from participants in a colonoscopy screening with a 5-marker blood test (CEA, anti-p53, osteopontin, seprase and ferritin) and found that the diagnostic performance for CRC of the 5-marker test was comparable with that of the guaiac-based fecal occult blood test (gFOBT), as they both identify 39% of all CRC cases at the specificity of 96% [22]. The results of our study support these recent results showing that combinations of serological biomarkers are valuable in the diagnosis of CRC. We found that the combined detection of CNPY2 isoform 2, CEA and CA19-9 resulted in a significantly better AUC than those of the individual tumor markers. Furthermore, the combination of these three markers resulted in an AUC of 0.786, which can be considered to indicate a high accuracy [23]. Based on the results mentioned above, the efficacy of a single biomarker for CRC detection is still limited, while the combination of various effective serological tumor markers or sequential use of them could achieve a better diagnostic efficiency for CRC.

As a rapid, high-throughput, quantitative immunoassay for the selective detection of target antigens, ELISAs offer considerable value in laboratory research applications and for the diagnosis of diseases on the basis of biomarkers [24,25]. Thus, the detection of CNPY2 isoform 2 was developed as a technique based on simple technical principles. In our study, a small volume of serum (only 200 µl) was required, which can be non-invasively obtained and easily stored, which contributed to better compliance by patients and allowed for convenient testing. As mentioned above, we found that serum CNPY2 isoform 2 presented a high diagnostic utility for distinguishing early-stage CRC patients from healthy controls. It has been demonstrated that the utilization and improvement of early CRC screening leads to a reduction in CRC incidence rates in developed countries [26]. Furthermore, the early diagnosis of CRC could improve prognoses and the quality of life of patients and ultimately reduce mortality due to CRC in the future [3,27]. However, effective screening programs for CRC mainly depend on the testing methods utilized. Based on our results, we encourage the integration of CNPY2 isoform 2 into routine non-invasive testing such as gFOBT and fecal immunochemical tests (FITs) for a further improvement in the efficiency of early CRC screening. Taken together, detection of CNPY2 isoform 2 could be easily applied in clinical practice and could even be effectively used for CRC screening.

Some limitations of the present study have to be considered. First, this retrospective study employed an uncontrolled methodology with a limited number of patients from a single institution; therefore, to validate these findings, a prospective study with a larger sample size from multiple centers is needed. Second, several disease conditions, such as infection, ischemia and diabetes mellitus, which may bias serum CNPY2 isoform 2 levels, could not be taken into consideration. In addition, we did not evaluate the value of serum CNPY2 isoform 2 for prognosis. Detection of changes in the serum concentration of CNPY2 isoform 2 might prove useful for dynamic monitoring of the prognosis of CRC patients who undergo surgical intervention. Nevertheless, our study is the first to suggest that the detection of serum CNPY2 isoform 2 could play a complementary role in the diagnosis of CRC, especially when combined with the detection of serum CEA and CA19-9.

## CONCLUSION

The ELISA technique is accurate and reproducible for the detection of CNPY2 isoform 2 in serum samples. Serum CNPY2 isoform 2 concentrations were significantly higher in CRC patients than in healthy controls. Combined detection of serum CNPY2 isoform 2, CEA and CA19-9 improved the diagnostic efficiency for CRC. Serum CNPY2 isoform 2 assays could be easily applied in clinical detection and this marker could even serve as a potential diagnostic biomarker in CRC screening.

## MATERIALS AND METHODS

### Subjects and sample selection

Blood samples from 430 CRC patients were collected from the Departments of Colorectal Surgery at Sun Yat-sen University Cancer Center between July 2004 and February 2015. All cases were staged according to the 2010 American Joint Committee on Cancer (AJCC) staging system. The inclusion criteria were as follows: (1) histologically confirmed adenocarcinoma; (2) single colorectal tumor; (3) no anti-cancer treatment before tumor resection; (4) and no other active malignancy. The blood samples were routinely obtained in the morning within 7 to 10 days before surgery. The 201 healthy controls were selected from the Department of Medical Examination of Sun Yat-sen University Cancer Center between January 2016 and May 2016; controls had no history of malignant disease or heart disease and had no infection at the time of examination. The blood samples from controls were also collected in the morning. Serum samples were separated by centrifuging the blood samples at 2000 to 2500 *g* for 10 min and stored at -80°C. The present study was performed according to the ethical standards of the World Medical Association Declaration of Helsinki. Informed consents were obtained from the patients and healthy controls before blood was drawn. The study was approved by the Institutional Research Ethics Committee of Sun Yat-sen University Cancer Center (Approval number: B2017-042-01).

### Generation of CNPY2 isoform 2 detection ELISA kit

Purified recombinant CNPY2 isoform 2 proteins and the monoclonal antibody clones were generated, and five effective monoclonal antibody clones were selected by using immunohistochemistry (IHC) as we previously reported [17]. Based on these five selected monoclonal antibody clones, a sandwich ELISA kit for CNPY2 isoform 2 was developed by Senboll Biotechnology Co., Ltd., using a pair of monoclonal antibodies. In brief, the first CNPY2 isoform 2 monoclonal antibody was pre-coated on 96-well plates used to bind the N-terminus of CNPY2 isoform 2 in blood samples. The second biotin-conjugated CNPY2 isoform 2 monoclonal antibody was used to interact with the C-terminus of CNPY2 isoform 2. In addition, streptavidin-horseradish peroxidase (HRP) and 3,3',5,5'-tetramethylbenzidine (TMB) substrates were also prepared. CNPY2 isoform 2 recombinant protein purified from E. coli. served as protein standard to calculate the concentration of CNPY2 isoform 2 protein in human serum. The stock solutions were stored at -80°C, and unopened kits were stored at 4°C.

### ELISA to quantify serum CNPY2 isoform 2

Serum CNPY2 isoform 2 was measured quantitatively by using the CNPY2 isoform 2 detection ELISA kit. After pipetting 100 μl of sample buffer to each well, 100 μl of CNPY2 isoform 2 standard stock solution and a serum sample were added to each wells. Subsequently, we covered the plate and incubated it at 4°C for 90 min. CNPY2 isoform 2 detection reagent was added to each well and incubated at 4°C for 60 min. The streptavidin-HRP working solution was added to each well, and then samples were incubated at 4°C for 60 min. After being washed, wells were developed with TMB, and the reaction was stopped by using a stop solution. Finally, the plates were read at 450 nm by a full-wavelength microplate reader (MD VersaMax, Molecular Devices, California, United States) in order to measure absorbance values.

### Immunoassays for CEA and CA19-9

The serum CEA and CA19-9 quantitative measurements were performed at the Department of Laboratory Medicine of Sun Yat-sen University Cancer Center. The concentrations of CEA and CA19-9 in the serum samples were determined by using the Elecsys CEA kit (catalog number: 11731629322, Roche Diagnostics GmbH, Mannheim, Germany) and Elecsys CA19-9 kit (catalog number: 11776193122, Roche Diagnostics GmbH, Mannheim, Germany) according to the manufacturer's instructions. The tested samples were subsequently assayed by an electrochemiluminescence analyzer (cobas E602, Roche, Germany). Abnormal reference values for CEA and CA19-9 were identified as >5.0 ng/ml and >35.0 U/ml, respectively.

### Statistical analysis

Comparisons of CNPY2 isoform 2 levels within the two groups were performed by using a Mann–Whiney U test, and differences between the groups were compared by using a Kruskal–Wallis H test. The predictive ability of CNPY2 isoform 2, CEA, CA19-9 and a combination of all 3 markers for CRC were determined by logistic regression. A receiver operating characteristic (ROC) curve analysis was applied to calculate the area under the ROC curve (AUC), 95% confidence interval (CI) and Youden’s index (sensitivity + specificity - 1) for each tumor marker. All the above-mentioned analyses were performed by using IBM SPSS statistics software, version 21.0 (IBM Corp., Armonk, NY, USA). The corresponding experimental figures were drawn using GraphPad Prism v6.0 software (GraphPad Software Inc, La Jolla, CA, USA). The statistical tests used above were two-sided, and a *P* value <0.05 was considered significant. Comparisons of two AUCs were conducted with DeLongs algorithm in the pROC package by using R software (version 3.2.0, The R Foundation, http://www.r-project.org). The *P* values were adjusted by Bonferroni correction, and the inspection level was 0.0167.

### Ethics approval and consent to participate

Study was approved by the Institutional Research Ethics Committee of Sun Yat-sen University Cancer Center (Approval number: B2017-042-01).The informed consents for using blood samples, before the initial treatment, were obtained from the patients.

### Availability of data and materials

The datasets analysed during the current study were available from the corresponding author on reasonable request. Anyone who is interested in the information should contact fangyj@sysucc.org.cn and wands@sysucc.org.cn.
